# Sequential Co-Immobilization of Enzymes on Magnetic Nanoparticles for Efficient l-Xylulose Production

**DOI:** 10.3390/ijms25052746

**Published:** 2024-02-27

**Authors:** Sanjay K. S. Patel, Rahul K. Gupta, Karthikeyan K. Karuppanan, In-Won Kim, Jung-Kul Lee

**Affiliations:** Department of Chemical Engineering, Konkuk University, Seoul 05029, Republic of Korea; sanjaykspatel@gmail.com (S.K.S.P.); guptarahul9m@gmail.com (R.K.G.); karthikk1529@gmail.com (K.K.K.); inwon@konkuk.ac.kr (I.-W.K.)

**Keywords:** co-immobilization, co-factor regeneration, l-arabinitol 4-dehydrogenase, l-xylulose, nicotinamide adenine dinucleotide oxidase, reusability, stability

## Abstract

Multi-enzymatic strategies have shown improvement in bioconversion during cofactor regeneration. In this study, purified l-arabinitol 4-dehydrogenase (LAD) and nicotinamide adenine dinucleotide oxidase (Nox) were immobilized via individual, mixed, and sequential co-immobilization approaches on magnetic nanoparticles, and were evaluated to enhance the conversion of l-arabinitol to l-xylulose. Initially, the immobilization of LAD or Nox on the nanoparticles resulted in a maximum immobilization yield and relative activity of 91.4% and 98.8%, respectively. The immobilized enzymes showed better pH and temperature profiles than the corresponding free enzymes. Furthermore, co-immobilization of these enzymes via mixed and sequential methods resulted in high loadings of 114 and 122 mg/g of support, respectively. Sequential co-immobilization of these enzymes proved more beneficial for higher conversion than mixed co-immobilization because of better retaining Nox residual activity. Sequentially co-immobilized enzymes showed a high relative conversion yield with broader pH, temperature, and storage stability profiles than the controls, along with high reusability. To the best of our knowledge, this is the first report on the mixed or sequential co-immobilization of LAD and Nox on magnetic nanoparticles for l-xylulose production. This finding suggests that selecting a sequential co-immobilization strategy is more beneficial than using individual or mixed co-immobilized enzymes on magnetic nanoparticles for enhancing conversion applications.

## 1. Introduction

Immobilization strategies have been widely studied to improve enzyme loading, residual activity, and stability, in order to overcome the significant limitations of stability and reuse associated with free enzymes [1,2]. The presence of His-tagged recombinant enzymes is helpful for easy purification and immobilization, especially in affinity-based systems [1,3]. Various enzymatic immobilization procedures have been demonstrated, including (i) encapsulation, (ii) adsorption on supports, (iii) covalent immobilization, (iv) cross-linking using linkers such as glutaraldehyde (GLA), or combinations of these methods, such as covalent immobilization followed by cross-linking [4,5,6,7]. Each method has its own advantages and disadvantages for the immobilization of certain enzymes. The key benefits of enzyme immobilization methods include (i) easy biocatalyst separation; (ii) abatement of downstream processing; (iii) superior recycling of biocatalysts; (iv) improved stability, relative to higher temperatures, pH, or solvents; and (v) feasibility for use with other enzymes via co-immobilization [5,6,7]. Significant disadvantages include (i) lower activity and reaction rates compared to free enzymes, (ii) surplus cost of immobilization supports, (iii) fouling, and (iv) disposal of exhausted immobilized biocatalysts through incineration or environmental issues due to material toxicity [1,4,8,9,10]. Such issues can potentially be solved by the selective use of immobilization methods, supports, and protein engineering-based approaches in specific enzyme immobilization [11]. In addition, multi-enzyme immobilization has proven to be more beneficial than free-enzyme mixtures for efficient bioconversion or bioremediation application [8,12,13,14]. As supports for enzyme immobilization, magnetic nanoparticles offer various advantages: (i) their magnetic properties allow for easy separation and recovery from reaction mixtures by using an external magnetic field; (ii) the large surface area of these nanoparticles provides a high enzyme loading capacity, allowing for more efficient immobilization and higher enzyme activity; and (iii) unique properties, such as superparamagnetism and high coercivity, which ensure the stability of immobilized enzymes during storage and reaction conditions. However, using magnetic nanoparticles as a support for enzyme immobilization also has several disadvantages, such as the high cost and complexity of synthesizing these nanoparticles and their potential for agglomeration or aggregation, which can affect the enzyme immobilization efficiency and reusability [15,16,17]. Enzyme inactivation due to interactions with the immobilization matrix or other enzymes in close proximity, difficulties in achieving optimal enzyme loading, and distribution on the immobilization support are other limitations of this immobilization system. Further research is thus needed to investigate the factors that contribute to the loss of enzyme activity during the co-immobilization process, which will develop methods to minimize this loss, and optimize enzyme loading and distribution on the immobilization support [13,15,17].

Rare sugars are monosaccharides that are rarely available or occur naturally in low abundance. These sugars and their derivatives play vital roles in various applications, including cosmetics, industry, and pharmaceutics [18,19,20,21]. In addition, these rare sugars can be used as precursors to synthesize high-value-added compounds. For example, l-xylulose is a rare ketopentose sugar used as a potent inhibitor of α-glucosidases and as an indicator of liver cirrhosis [22,23]. It can be used for the synthesis of (2S,3S,4R)-1-O-(α-D-galactopyranosyl)-N-hexacosanoyl-2-amino-1,3,4-octadecanetriol, an antitumor and immunostimulatory agent [24,25]. The enzymatic production of l-xylulose from l-arabinitol has been studied; however, the process is primarily governed by costly cofactors, such as nicotinamide adenine dinucleotide (NADH/NAD^+^), which are an integral part of it [26,27]. Therefore, l-xylulose production using a cofactor regeneration system is necessary for efficient conversion. In previous studies, the individual His-tagged protein immobilization of NADH oxidase (Nox) from *Streptococcus pyogenes* and L-arabinitol 4-dehydrogenase (LAD) from *Hypocrea jecorina* was performed by encapsulation [10]. The covalent co-immobilization of enzymes on magnetic nanoparticles has been proven to be beneficial for achieving higher stability and structural robustness over encapsulation (which is primarily limited by the structural integrity of the immobilized system, namely metal-protein hybrids), especially for recycling and easy separation [28,29,30,31]. 

The present study demonstrates various immobilization strategies for LAD and Nox via individual, mixed, and sequential methods on magnetic nanoparticles that are used to develop an efficient system for converting l-arabinitol to l-xylulose. Sequential co-immobilized enzymes exhibited better stability and more efficient recycling for converting l-arabinitol to l-xylulose under cofactor regeneration conditions than a free enzyme mixture. These findings suggest that the sequential co-immobilization of His-tagged recombinant enzyme systems on magnetic particles over mixed co-immobilized and free enzymes is a promising approach for industrial bioconversion applications. 

## 2. Results and Discussion

### 2.1. Immobilization of LAD or Nox on Magnetic Nanoparticles

**Figure 1:**
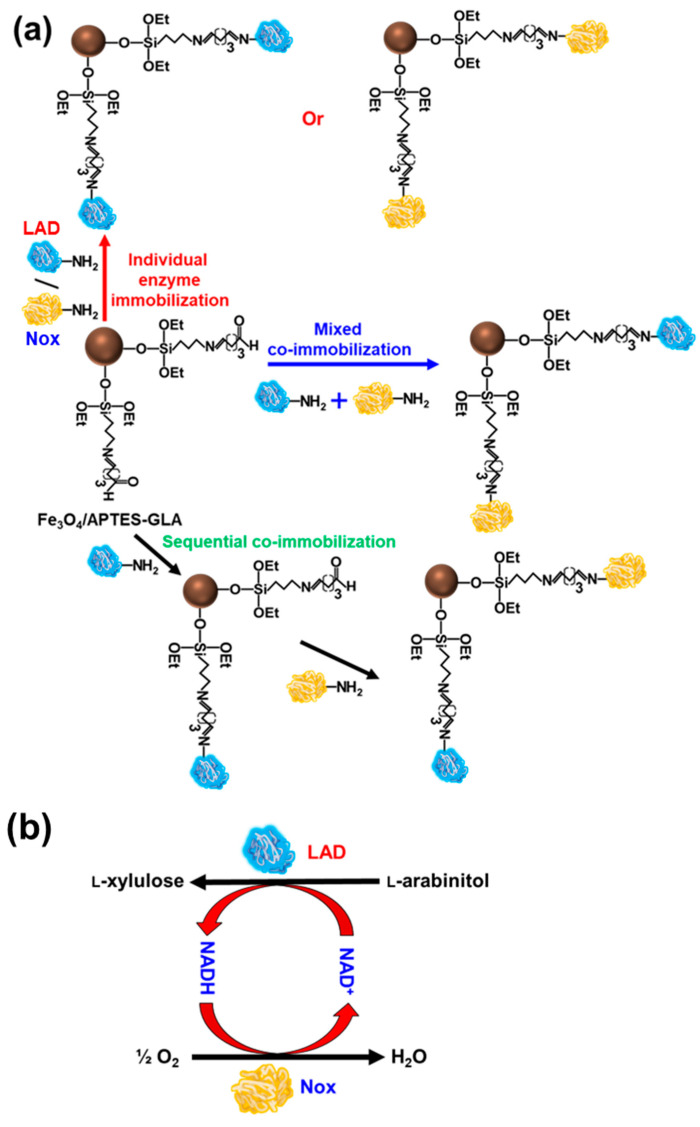
A schematic illustration of individual or mixed and sequential co-immobilization of l-arabinitol-4-dehydrogenase (LAD) and NADH oxidase (Nox) on functional activated magnetic (Fe_3_O_4_) nanoparticle (**a**); and (**b**) conversion of l-arabinitol to l-xylulose by LAD and Nox under cofactor regeneration conditions.

Initially, the immobilization of individual enzymes was assessed to elucidate the suitability of the supports as pure or composite magnetic nanoparticles and their functional activation. In the general considerations of enzyme immobilization, regardless of the immobilization procedure adapted, activity recovery, enzyme loading, immobilization efficiency, immobilization yield (IY), composite properties (enzyme + support), (i.e., size and density), hydrophobicity, and mechanical robustness are imperative in the selection of reactor configuration and downstream processing applications [15]. The terminology for calculating immobilization is highly variable in the literature. The success of enzyme immobilization is determined by measuring the IY, immobilization efficiency, and activity recovery [5,15]. The immobilization of LAD or Nox was performed on magnetic nanoparticles functionally activated with GLA, 3-aminopropyltriethoxysilane (APTES), and APTES-GLA at 4 °C for 24 h (Table 1). The IY and relative activity (RA) of LAD on functionalized Fe_3_O_4_ nanoparticles varied in the range of 48.5–91.4% and 67.6–98.8%, respectively. In contrast, the control Fe_3_O_4_ nanoparticles without functional activation resulted in a much lower IY and RA, of 10.2% and 23.4%, respectively, for LAD. Nox immobilization on functionally-activated Fe_3_O_4_ nanoparticles resulted in a high IY and RA of 92.1% and 97.2%, respectively. Previously, glycerol dehydrogenase and Nox, individually immobilized on microporous resins, exhibited low IY and RA values of up to 70% and 40%, respectively [32]. The immobilization of enzymes on the magnetic composite SrFe_12_O_19_ resulted in IY and RA values, of up to 84.6% and 86.8% for LAD, and up to 83.5% and 90.1% for Nox, respectively. In contrast, without functional activation, SrFe_12_O_19_ nanoparticles showed low IY and RA for LAD and Nox, in the range of 8.1–9.5% and 18.7–19.2%, respectively. These results suggest that the functional activation of nanoparticles by various groups can be significantly altered by IY and RA, which has also been described for other enzymes [5,33]. The maximum IY and RA for LAD and Nox were recorded for the nanoparticles functionally activated by APTES, followed by GLA. Here, covalent immobilization appeared to be efficient for the immobilization of both LAD and Nox. Further, the leaching of co-immobilized enzymes on Fe_3_O_4_/APTES-GLA was analyzed by treatment with 1 M of NaCl for 1 h of incubation at 25 °C, to measure the association of hydrophobic and ionic interactions during immobilization [5]. No protein was detected in the supernatant, which confirmed the purely covalent immobilization of these enzymes on Fe_3_O_4_/APTES–GLA [5,34]. The spherical shape and size of nanoparticles, including magnetic supports, highly influenced enzyme immobilization properties such as IY and RA [17,35,36]. Previously, the covalent immobilization of LAD on various supports, including Anberlites, Eupergit, Duolite, and multi-wall carbon nanotubes, showed a lower IY than that of Fe_3_O_4_ nanoparticles [36]. In contrast, Nox immobilization on agarose, Purolite, and Eupergit showed a much lower RA in the range of 21–37% [37]. On the basis of the high IY and RA results, the Fe_3_O_4_/APTES–GLA nanoparticles, or those immobilized with enzymes, were used for further studies. The immobilization strategies for LAD and Nox are shown in Figure 1. For covalent enzyme immobilization to Fe_3_O_4_-APTES/GLA, the interaction between amino groups of the amino acids (i.e., lysine of the enzyme and GLA-activated groups of Fe_3_O_4_ nanoparticles) resulted in the formation of covalent bonds.

### 2.2. Characterization of Immobilized LAD or Nox on Fe_3_O_4_ Nanoparticles

**Figure 2:**
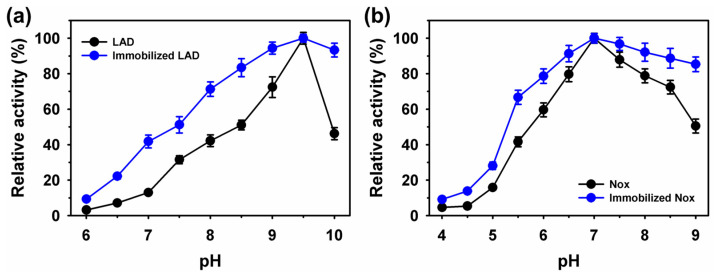
Activity profiles of free and immobilized enzyme on magnetic Fe_3_O_4_ nanoparticles at various pH for (**a**) LAD, and (**b**) Nox. The free and immobilized activity of 87.2 and 86.1 µmol min^−1^ mg protein^−1^ for LAD, and 335 and 326 µmol min^−1^ mg protein^−1^ for Nox, was considered as 100%, respectively.

Immobilization of enzymes is considered a beneficial approach to achieve higher bioprocess stability over a broad pH and temperature range, compared to their free form [36,37,38]. The activity profiles of LAD and Nox at various pH values are shown in Figure 2.

**Figure 3:**
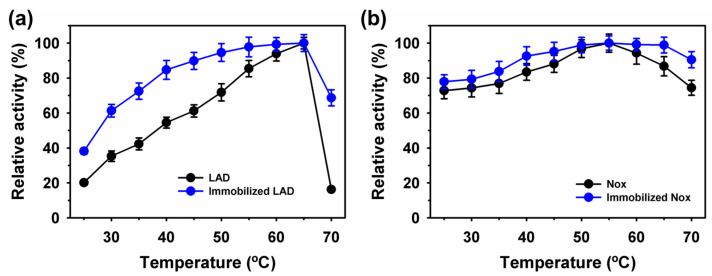
Activity profiles of the free and immobilized enzyme on magnetic Fe_3_O_4_ nanoparticles at various temperatures for (**a**) LAD, and (**b**) Nox. The free and immobilized activity of 374 and 238 µmol min^−1^ mg protein^−1^ for LAD, and 335 and 326 µmol min^−1^ mg protein^−1^ for Nox, was considered as 100%, respectively.

The optimum pH of 9.5 and 7.0, respectively, were observed for free and immobilized enzymes. After immobilization, the enzymes showed a broad pH profile. Immobilized LAD exhibited 2.9- (9.3%) and 2.0-fold (93.3%) higher residual activity, at pH 6.0 and 10.0, over the free enzyme, with the value of 3.2% and 46.2%, respectively. Similarly, immobilized Nox showed 2.0- and 1.7-fold higher residual activity at pH values of 4.0 and 9.0, respectively, than the free enzyme, with activity of 4.6% and 50.5% at the corresponding pH values, respectively. A similar optimum temperature for the free and immobilized enzymes was noted at 65 °C for LAD, and 55 °C for Nox (Figure 3). 

After immobilization on Fe_3_O_4_, the enzymes exhibited better residual activity profiles at temperature ranges of 25–70 °C. At a high temperature of 70 °C, LAD and Nox showed 4.2- (68.7%) and 1.2-fold (90.5%) higher residual activity than their corresponding free forms, with values of 16.2% and 74.4%, respectively. Property changes, including in activity profiles and enzyme stability, after immobilization can be linked to secondary structure alteration [15,39]. In secondary structure analysis, an increase was observed in the LAD α-helix content, from 39.1–46.9; in β-sheet, from 31.6–22.3%; in β-turn, from 12.1–20.4%; and in others, from 17.2–10.4% (Table S1). Further changes were found in the Nox α-helix content, from 60.7–71.3%; in β-sheets, from 10.5–6.2%; in β-turns, from 7.9–10.2; and in others, from 20.9–12.3%. These observations suggest that LAD or Nox immobilization led to secondary structural alterations. The kinetic parameters (*V*_max_ and *K*_m_) of the free and immobilized enzymes were determined using the Michaelis-Menten model, as presented in Table 2. The apparent *V*_max_ and *K*_m_ values for immobilized LAD were 92.2 µmol min^−1^ mg protein^−1^ and 15.5 mM, compared to free enzyme values of 93.2 µmol min^−1^ mg protein^−1^ and 17.8 mM, respectively. Previously, LAD immobilized on multiwall carbon nanotubes and silica nanoparticles resulted in up to 42.2% lower affinity towards the substrate [36]. The decline in *K*_m_ after enzyme immobilization on silica nanoparticles was associated with strong binding of the enzyme to the support, resulting in a lower affinity towards the substrate [36,38]. The *V*_max_ and *K*_m_ values for immobilized Nox were 330 µmol min^−1^ mg protein^−1^ and 23.9 µM, respectively, with values of 340 µmol min^−1^ mg protein^−1^ and 27.1 µM, respectively, for the free enzyme. After immobilization, the catalytic efficiency (*K*_cat_/*K*_m_) improved from 210 to 240 mM^−1^ min^−1^ for LAD, and from 630 to 690 µM^−1^ min^−1^ for Nox.

### 2.3. Mixed and Sequential Co-Immobilization of LAD and Nox on Fe_3_O_4_ Nanoparticles

The immobilization of LAD and Nox on Fe_3_O_4_/APTES–GLA exhibited better RA and stability profiles. The mixed and sequential co-immobilization of LAD and Nox was evaluated after immobilization on Fe_3_O_4_/APTES–GLA nanoparticles. A schematic diagram of the mixed and sequential enzyme immobilization on nanoparticles and l-xylulose production under cofactor regeneration conditions is shown in Figure 1. The co-immobilization of LAD and Nox at a 1:3 ratio was performed based on better conversion to their other ratios of 1:2 and 2:1 (Table S2). The mixed co-immobilization of enzymes on Fe_3_O_4_/APTES–GLA nanoparticles resulted in IYs of 87.2% (87.2 mg) for LAD and 83.8% (26.8 mg) for Nox, with a total protein loading of 114 mg per g of support. In the sequential co-immobilization of enzymes, the immobilization of Nox at the second stage on the initially immobilized LAD (IY and RA of 91.4% and 98.8%, respectively) showed an IY of 96.0%, with an overall loading of 122 mg (91.4 mg of LAD and 30.7 mg of Nox) per g of Fe_3_O_4_/APTES–GLA nanoparticles (Table 3). Here, a minor alteration in the conversion efficiency of l-arabinitol to l-xylulose by mixed and sequential co-immobilization might be associated with different extents of steric hindrance in these co-immobilization procedures [15,35]. Previously, a lower IY of 83% was reported for xylose dehydrogenase (XDH) and alcohol dehydrogenase (ADH) co-immobilized at a ratio of 2:1 on magnetic silica nanoparticles [40]. Fe_3_O_4_@SiO_2_–NH_2_ nanoparticles showed a low loading of 28 mg/g of support for the co-immobilization of glucose dehydrogenase and (R)-1-phenylethanol dehydrogenase [41]. In contrast, multi-enzyme (Endo-1,4-β-D-glucanase, xylanase, and amylase) immobilization in a spherical shape with a size of 25 nm Fe_3_O_4_ nanoparticles-based synthesized magnetic composite offers a lower loading of 14.7 mg of soluble protein/g of support [35]. One advantage of immobilized enzymes is that their application offers minor risk of product contamination with negligible allergenicity [15].

### 2.4. Analysis of Co-Immobilized Enzymes on Fe_3_O_4_ Nanoparticles

**Figure 4:**
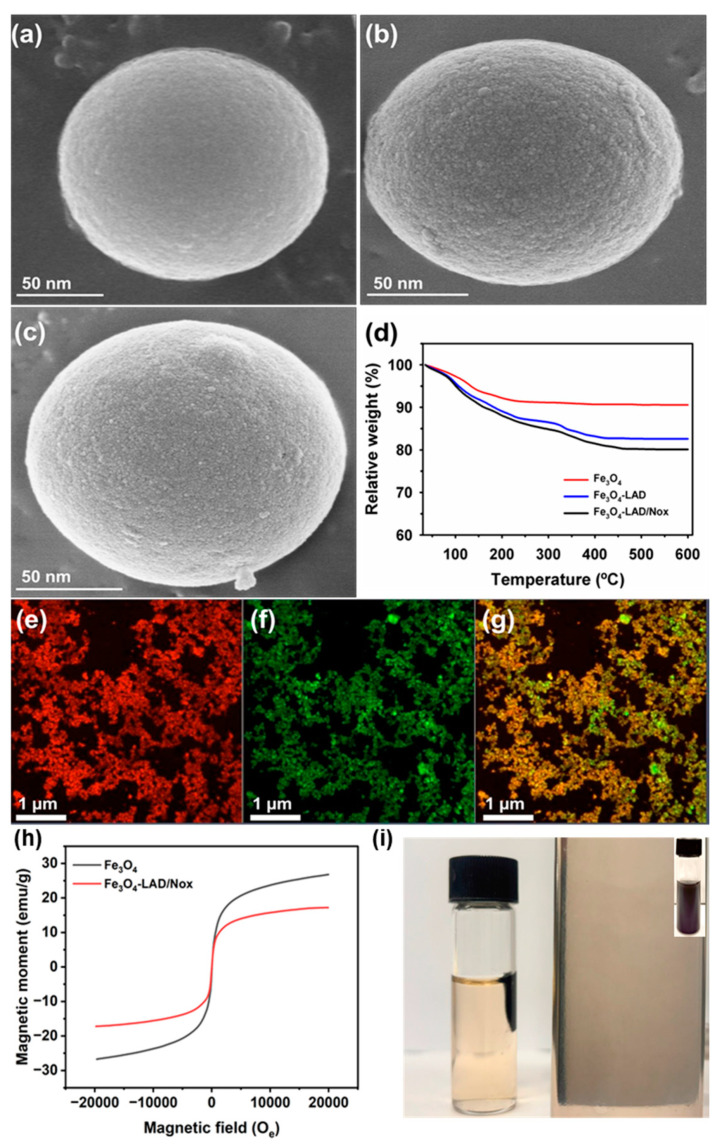
Field-emission-scanning electron microscopy analysis of (**a**) magnetic Fe_3_O_4_ nanoparticles, (**b**) immobilized with LAD, (**c**) after sequential co-immobilization of Nox on immobilized LAD, (**d**) thermogravimetric and confocal laser scanning microscopy analysis of sequential co-immobilized enzymes of (**e**) rhodamine-B-isothiocyanate-labeled LAD, (**f**) fluorescein-isothiocyanate-labeled Nox, and (**g**) merged images of (**e**,**f**), and magnetic property measurement of Fe_3_O_4_ nanoparticles immobilized with enzymes (**h**), and separation in the presence of external magnetic field (**i**).

Field-emission-scanning electron microscopy (FE-SEM) was used to validate the sequential co-immobilization of enzymes on Fe_3_O_4_ nanoparticles (Figure 4a–c). The immobilization of LAD followed by Nox resulted in a rough texture, compared to the smoother surface of the pure nanoparticles, confirming enzyme immobilization. Subsequently, thermogravimetric analysis (TGA) was performed to validate sequential enzyme immobilization (Figure 4d). The immobilization of LAD on Fe_3_O_4_ nanoparticles resulted in approximately 17.4% weight reduction, compared with 9.3% for pure nanoparticles. Furthermore, the immobilization of Nox resulted in a 19.9% weight reduction, confirming the sequential co-immobilization of enzymes on magnetic nanoparticles. To validate the co-immobilization of LAD and Nox on magnetic nanoparticles, fluorescently-labeled LAD with rhodamine B isothiocyanate (RBITC) and Nox with fluorescein isothiocyanate (FITC) were used for sequential co-immobilization [10]. In the confocal laser scanning microscopy (CLSM) analysis, the visualization of red and green fluorescence of RBITC and FITC for LAD and Nox, respectively, established the co-immobilization of the enzymes (Figure 4e–g). The super-paramagnetic properties of the Fe_3_O_4_ particles were compared before and after enzyme immobilization (Figure 4h). The nanoparticles showed a magnetization saturation of 26.8 electromagnetic unit g^−1^ (emu g^−1^), whereas those immobilized with enzyme showed a value of 17.2 emu g^−1^. This change may correlate with enzyme binding to the magnetic nanoparticles [17]. The magnetic separation of the immobilized enzyme is shown in Figure 4i. Binding of the enzymes to the nanoparticles was confirmed by Fourier-transform infrared (FTIR) analysis (Figure S1). The characteristic common peaks at 530–600, 950–1020, 1620–1710, 2800–3000, and 3200–3400 cm^−1^ established the occurrence of Fe-O, Si-O-Si, C=O (aldehyde), -CH_2_/-CH_3_, and -OH groups, respectively. The presence of these groups confirmed the functionalization of Fe_3_O_4_ nanoparticles [42,43]. The presence of typical peaks of peptide bonds at 1550–1640 cm^−1^ for C=O stretching (amide I, 1640 cm^−1^) and N–H bending (amide II, 1550 cm^−1^) validated the enzyme immobilization on nanoparticles. Further, the rough texture of Fe_3_O_4_-APTES/GLA nanoparticles, compared to that of the control in high-resolution transmission electron microscopy (HR-TEM) images, validates the enzyme immobilization (Figure S2).

### 2.5. Production of l-Xylulose by Free and Immobilized Enzymes on Fe_3_O_4_ Nanoparticles

**Figure 5:**
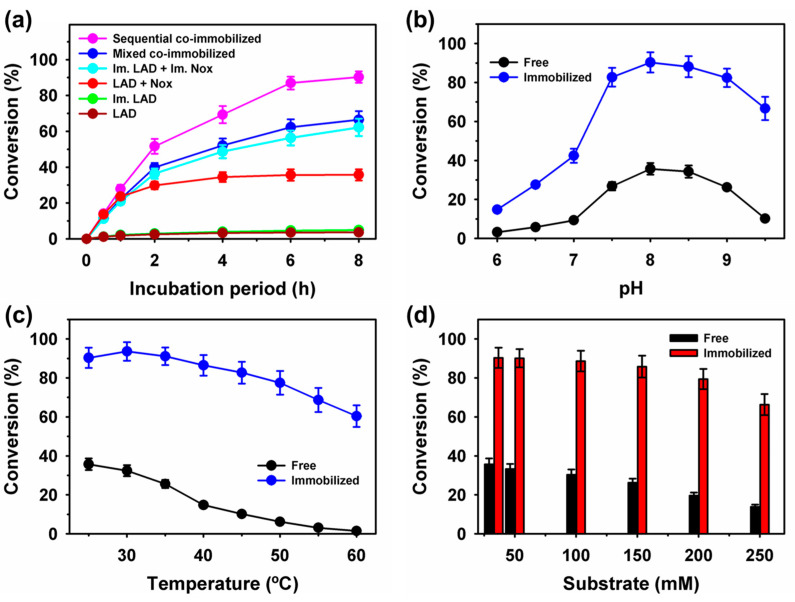
Production of l-xylulose by free, individual, and co-immobilized enzymes time profile (**a**); and the influence of (**b**) pH, (**c**) temperatures, and (**d**) substrate concentration on the conversion of l-arabinitol to l-xylulose by free and sequential co-immobilized LAD and Nox.

Free and immobilized LAD resulted in l-arabinitol to l-xylulose conversions of 3.6% and 4.9%, respectively, after 8 h of incubation at pH 8.0 (Figure 5a). These findings suggest that enzyme immobilization improved conversion by approximately 37%. Furthermore, supplementation with Nox can enhance conversions via cofactor regeneration. Conversion by free and immobilized enzyme mixtures of LAD and Nox showed maximum conversions of 35.7% and 62.2%, respectively (Figure 5a). Co-immobilization of enzymes has been proven to be a beneficial approach for improving conversion because of their proximity to the scattered behavior of free forms of enzymes in the reaction mixture and better enzyme stabilization after immobilization [44,45]. Therefore, possible combinations of LAD and Nox via mixed and sequentially co-immobilized systems were employed for conversion and exhibited better conversion yields of 66.5% and 90.3%, respectively (Figure 5a). In the presence of free and co-immobilized Nox, the enhancement was 9.9- and 25.1-fold higher, respectively, than that of free LAD. Overall, under the cofactor regeneration condition, a significant increase in the total turnover number of up to 153-fold was observed for a sequentially co-immobilized system, compared to free LAD alone as a control (Table 3). Earlier, a lower enhancement of up to 1.6-fold was reported for the co-immobilization of enzymes by mixing through metal-protein hybrids to produce D-tagatose as a rare sugar [21]. The influence of pH on l-xylulose production by sequentially co-immobilized enzymes (LAD and Nox) and the corresponding free enzyme mixture was initially evaluated. An optimum pH of 8.0 was noted for the free and co-immobilized enzymes (Figure 5b). Remarkably, the co-immobilized enzymes exhibited 4.6- and 6.5-fold higher l-xylulose production at pH of 6.0 and 9.5 than the free enzyme mixture, with conversion values of 3.2% and 10.2%, respectively. Previously, an approximately 1.2-fold improvement in the conversion stability of xylose to xylonic acid was reported for co-immobilized xylose dehydrogenase and alcohol dehydrogenase on magnetic silica nanoparticles at different pH and temperature values, compared to free enzymes [40]. Free enzyme mixture exhibited a conversion of 35.7% at 25 °C; subsequently increasing the reaction temperature to 60 °C significantly decreased the conversion to 1.5% (Figure 5c). A superior optimum temperature of 30 °C was noted for sequentially co-immobilized LAD and Nox with a maximum conversion of 93.6%; at 60 °C immobilized enzymes showed 40-fold higher stability for l-xylulose production. Furthermore, the substrate concentration increased from 32.8 to 250 mM, and the l-xylulose production yield declined from 35.7% to 13.8% for the free enzyme mixture (Figure 5d). Interestingly, the co-immobilized enzymes maintained a high conversion rate of 66.3% at a substrate concentration of 250 mM.

### 2.6. Storage Stability, Reusability and Leaching Measurements

**Figure 6:**
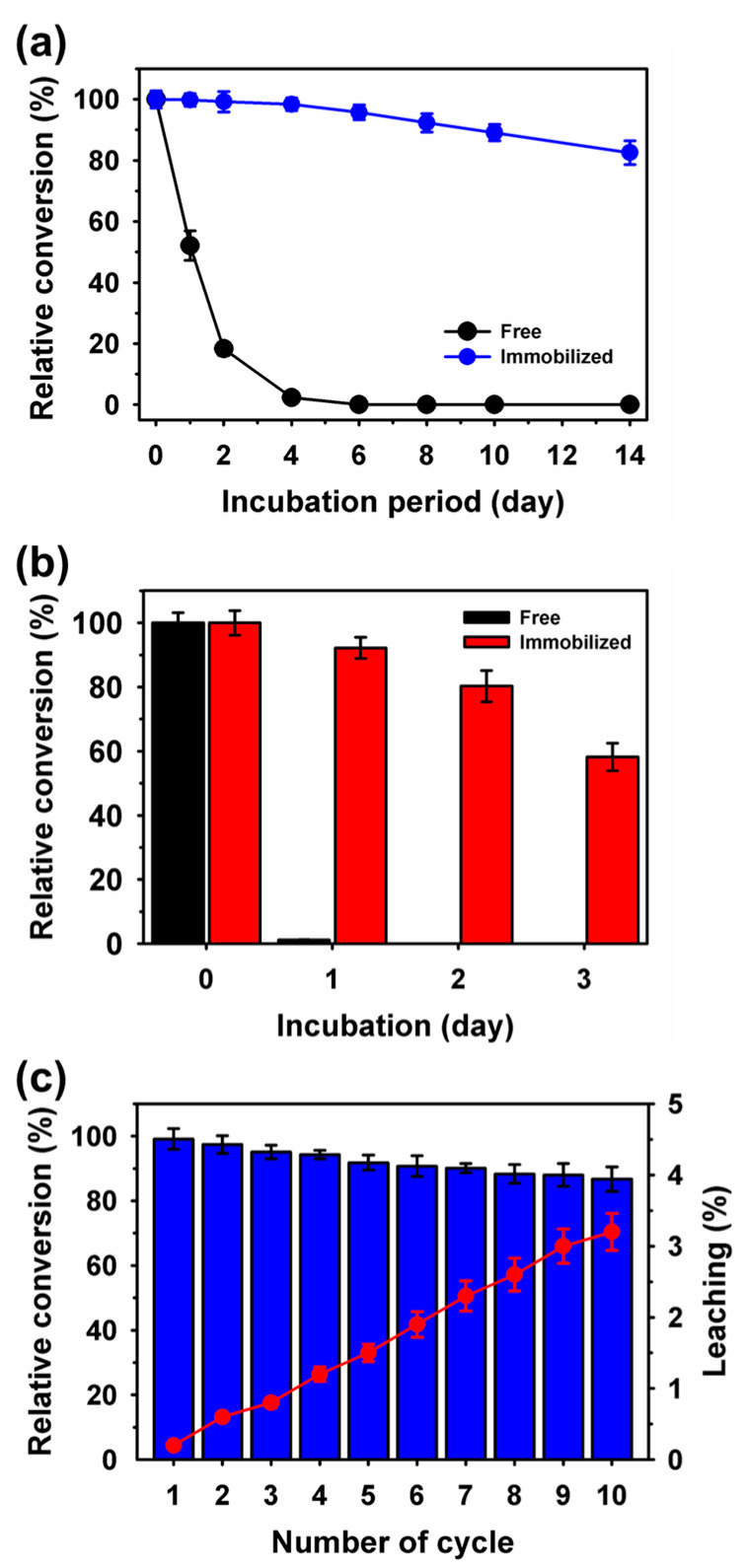
Production stability of l-xylulose by free and co-immobilized enzymes at (**a**) 4 °C, and (**b**) 25 °C, and (**c**) repeated batch recycling and leaching.

Immobilized enzymes are essential for better rare sugar conversion and recycling [41,46,47,48]. The storage stabilities of free and co-immobilized enzymes were compared at 4 °C and 25 °C (Figure 6a,b). Initially, at 4 °C, the free enzymes showed almost entirely declined conversion after an incubation of 4 d. However, under similar conditions, the co-immobilized enzymes showed a high relative conversion of 98.4%. After 14 d of incubation, the co-immobilized enzymes exhibited a residual conversion of 82.5%. At room temperature, within a day of incubation, the free enzyme mixture lost conversion, whereas the co-immobilized enzymes retained high stability, with a residual conversion yield of 58.2% within three days of incubation.

Previously, an approximately three-fold storage stability improvement was reported for XDH and ADH co-immobilized on magnetic-silica nanoparticles, compared to free enzymes after incubation for 20 d [40]. Production of l-xylulose was analyzed under repeated batch conditions in a 5 mL thermoreactor with a stir bar for 10 cycles. The reaction mixture contained 250 mM of l-arabinitol at 25 °C. During the initial cycle of incubation for 4 h, a 57.8% conversion was achieved. After 10 reuse cycles, the co-immobilized enzymes exhibited a high conversion of 50.1%, which was equivalent to a relative conversion of 86.7%. Previously, >35% of residual activity was lost within five cycles of reuses for co-immobilized XDH and ADH on magnetic-silica nanoparticles [40]; there was also 50% reduction of D-tagatose as rare sugar production by co-immobilized enzymes as metal-protein hybrids-based system [21]; and declined residual activity of up to 60% for co-immobilized glucoamylase and α-amylase on pure magnetic nanoparticles [38]. In addition, co-immobilization of glucose dehydrogenase and (R)-1-phenylethanol dehydrogenase on Fe_3_O_4_@SiO_2_–NH_2_ nanoparticles resulted in an approximately 30% residual activity after eight cycles of reusability [41]. To confirm the decline in conversion during successive cycles, enzyme leaching was evaluated by measuring the total protein concentration in the supernatant, and a cumulative leaching of 3.2% of the enzymes during recycling was observed. This suggests that enzyme inactivation was the primary cause of the decline in conversion.

## 3. Materials and Methods

### 3.1. Materials and Reagents

Fe_3_O_4_ and SrFe_12_O_19_ nanoparticles, APTES, l-arabinitol, GLA, NADH, NAD^+^ were purchased from Sigma-Aldrich (St. Louis, MO, USA). All other reagents were of analytical or biotechnological grade.

### 3.2. Cell Culture and Protein Purification

*Escherichia coli* BL21 harboring pET28a-LAD (*H. jecorina*) or pET28a-Nox (*S. pyogenes*) was cultivated in Luria–Bertani medium containing 50 µg/mL of kanamycin at 37 °C. The expression of LAD and Nox was induced by 0.1 mM of isopropyl-β-d-thiogalactopyranoside. Furthermore, cells were disrupted by sonication at 4 °C for 5 min, and the lysate was centrifuged at 10,000× *g* for 20 min (at 4 °C) to remove the cell debris. The resulting crude extract was purified using a Ni-NTA Super-flow column (3.4 × 13.5 cm, QIAGEN, Hilden, Germany). The purified enzyme fractions were analyzed by sodium-dodecyl-sulfate-polyacrylamide gel electrophoresis (12%) and visualized using Coomassie blue R250 (Figure S3).

### 3.3. Functionalization of Nanoparticles and Immobilization of LAD or Nox

The nanoparticles (200 mg) were washed and functionalized with GLA (1 M) for 2 h at room temperature to provide aldehyde groups on their surfaces, as previously described [6]. APTES activation of nanoparticle surfaces was evaluated in toluene supplemented with APTES (2%, *v*/*v*) at room temperature for 12 h [33]. For combined functional activation, the nanoparticles were functionalized with APTES, followed by GLA. The particles were then washed thoroughly with distilled water and stored at room temperature. For enzyme immobilization, 10 mg of modified nanoparticles were mixed with 1 mg of LAD in a phosphate buffer (100 mM, pH 7.0) for 24 h at 4 °C. Unbound enzymes were separated in the supernatant by centrifugation for 10 min at 10,000× *g* (4 °C). The protein concentration in the supernatant was measured using the Bradford method [6]. The IY and RA were calculated as follows:

IY (%) = ratio of immobilized enzyme to added enzyme × 100,(1)

RA (%) = ratio of immobilized enzyme to free enzyme activity × 100.(2)

### 3.4. Activity Measurements

The activity of purified enzymes was determined spectrophotometrically by monitoring the change in A340 upon reduction and oxidation of NAD^+^, and NADH for LAD and Nox at 25 °C, respectively [10]. The LAD assay mixture for oxidation consisted of NAD^+^ (1.5 mM), l-arabinitol (200 mM), and the enzyme in 100 mM Tris-glycine-NaOH (pH 9.5). The Nox assay mixture consisted of NADH (200 μM) and the enzyme in 50 mM potassium-phosphate buffer (pH 7.0). One unit of enzyme activity was defined as the amount of enzyme required to produce 1 μmol of NADH or NAD^+^ per minute for LAD or Nox under the assay conditions.

### 3.5. Characterization of Immobilized LAD or Nox 

Initially, the activity profiles of free and immobilized LAD or Nox were compared in buffers (50 mM of sodium-acetate, pH 4.0–5.5; potassium-phosphate, pH 6.0–7.5; tris-HCl-NaOH, pH 8.0–8.5; and glycine-NaOH, pH 9.0–10.0). Next, the influence of temperature was analyzed by assaying the enzyme samples over the range of 25–70 °C. Finally, the kinetic parameters *K*_m_, *V*_max,_ and catalytic efficiency (*K*_cat_/*K*_m_) were measured using l-arabinitol (25–400 mM) and NADH (5–400 mM) for free and immobilized LAD and Nox, respectively [10]. Kinetic parameter values for the substrates l-arabinitol or NADH were obtained through nonlinear regression analysis using GraphPad Prism 5 (San Diego, CA, USA).

### 3.6. Mixed and Sequential Co-Immobilization of LAD and Nox on Fe_3_O_4_ Nanoparticles for l-Xylulose Production 

For the mixed co-immobilization of LAD and Nox of Fe_3_O_4_/APTES-GLA nanoparticles, 10 mg of modified nanoparticles were mixed with 1 mg of LAD and 0.32 mg of Nox in a phosphate buffer (100 mM, pH 7.0) for 24 h at 4 °C. To perform sequential co-immobilization, Fe_3_O_4_/APTES–GLA nanoparticles initially immobilized with LAD (IY of 90.4%) were used to immobilize Nox at the second stage with the addition of 0.32 mg of the enzyme to the phosphate buffer (100 mM, pH 7.0) for 24 h at 4 °C. The l-xylulose production by free and co-immobilized enzymes was evaluated using l-arabinitol (32.8 mM), free enzyme mixture (0.33 mg of LAD and 0.11 mg of NOx), mixed co-immobilized enzymes (3.9 mg), sequential co-immobilized enzymes (3.6 mg), and NAD^+^ (2 mM) for 8 h. Subsequently, the l-xylulose production by free and co-immobilized enzymes was compared at different pH (6.0–9.5), temperatures (25–60 °C), and l-arabinitol concentrations (32.8–250 mM). 

### 3.7. Storage Stability and Reusability of Sequential Co-Immobilized Enzymes on Fe_3_O_4_ Nanoparticles for l-Xylulose Production 

The storage stability of free and co-immobilized enzymes was assessed for conversion of l-arabinitol to l-xylulose using l-arabinitol (32.8 mM), free enzyme mixture (0.33 mg of LAD and 0.11 mg of NOx), co-immobilized enzymes (3.6 mg), and NAD^+^ (2 mM) at 4 °C and 25 °C for 30 d. Reusability for up to 10 cycles was analyzed under repeated batch conditions for l-xylulose production in a 5 mL thermoreactor containing 250 mM substrate and NAD^+^ (2 mM) at pH 8.0 with incubation of 4 h. After each cycle, the co-immobilized enzyme was recovered by centrifugation and used as a biocatalyst in the next process. The initial conversion rate was considered 100%. Furthermore, the leaching (%) of enzymes was measured as the ratio of total protein content in the supernatant to the initial enzymes bound on the nanoparticles × 100 [6]. 

### 3.8. Instrumental Measurements

Catalytic absorption spectra were measured using a spectrophotometer (6705, Jenway Scientific, Stone, UK). For secondary structure examination, the circular dichroism (CD) analysis was done by a CD detector (Chirascan-plus, Applied Photophysics, Leatherhead, UK). FE–SEM was performed using a JSM-6700F microscope (JEOL, Tokyo, Japan) and HR–TEM using JEM-2100F (JEOL, Tokyo, Japan). The decomposition characteristics of pure magnetic nanoparticles immobilized with enzymes were analyzed by TGA (Seiko Exstar 6000 TG/DTA 6100, Chiba, Japan). Fluorescence labeling of LAD and Nox, with RBITC and FITC, respectively, was performed to validate the co-immobilization of enzymes with CLSM (ZEISS LSM900, Baden-Württemberg, Germany), as described previously [10]. The magnetic properties of the particles were measured using a SQUID/VSM magnetometer (MPMS^®^3, San Diego, CA, USA) at 298 K. The l-xylulose content was determined using high-pressure liquid chromatography [10]. All experimental values are based on three replicates.

## 4. Conclusions

In conclusion, this study reports the immobilization of LAD and Nox on functionally activated magnetic nanoparticles via individual, mixed, and sequential co-immobilization approaches to produce l-xylulose from l-arabinitol. Initially, the functionalization of nanoparticles resulted in a high IY and RA of LAD and Nox and improved their biocatalytic profiles at various pH and temperatures. Furthermore, co-immobilization of these enzymes via a mixed and sequential strategy demonstrated significantly higher production of l-xylulose than free and individually-immobilized systems in the cofactor regeneration system. Sequential immobilization of these enzymes on Fe_3_O_4_ nanoparticles remarkably enhanced the conversion of l-arabinitol to l-xylulose and stability, compared to free enzyme mixtures. In addition, the co-immobilized system retained a high recycling potential under repeated batch conditions over ten cycles of reusability. A competitive hindrance in the availability of a free functional group for the second enzyme immobilization (in a sequential procedure or partial enzyme inactivation via interactions with the immobilization matrix) highlights a limitation that might be associated with this system. This finding suggests that selecting a co-immobilization strategy can significantly influence enzyme properties and stability for higher conversion, and can also be used for other multi-enzymatic systems for broad industrial applications.

## Figures and Tables

**Table 1:** Individual immobilization of enzymes on various functionalized magnetic nanoparticles.

Nanoparticles	LAD		Nox	
	Immobilization Yield (IY, %)	Relative Activity (RA, %)	IY %	RA (%) ^a^
Fe_3_O_4_	10.2 ± 0.8	23.4 ± 1.9	8.6 ± 1.2	20.8 ± 1.6
Fe_3_O_4_GLA	85.6 ± 4.8	95.3 ± 6.5	87.3 ± 3.7	92.9 ± 7.8
Fe_3_O_4_APTES	48.5 ± 4.4	67.6 ± 4.9	57.2 ± 4.6	71.3 ± 6.6
Fe_3_O_4_/APTES-GLA	91.4 ± 4.8	98.8 ± 7.2	92.1 ± 3.8	97.2 ± 7.0
SrFe_12_O_19_	9.5 ± 0.8	18.7 ± 1.5	8.1 ± 0.7	19.2 ± 1.6
SrFe_12_O_19_/GLA	73.2 ± 6.1	85.4 ± 6.4	74.8 ± 1.2	82.4 ± 6.9
SrFe_12_O_19_/APTES	52.1 ± 3.7	60.5 ± 4.4	48.7 ± 4.2	65.1 ± 5.4
SrFe_12_O_19_/APTES-GLA	84.6 ± 4.9	86.8 ± 4.8	83.5 ± 5.3	90.1 ± 6.9

^a^ The activity of 87.2 and 335 µmol min^−1^ mg protein^−1^ for LAD and Nox was considered as 100%, respectively.

**Table 2:** Kinetic parameters of free and immobilized l-arabinitol-4-dehydrogenase (LAD) or NADH oxidase (Nox) on magnetic Fe_3_O_4_/APTES–GLA nanoparticles.

Enzyme	*V*_max_ (µmol min^−1^ mg protein^−1^)	*K*_m_	*K*_cat_/*K*_m_
LAD	93.2 ± 7.7	17.8 ± 2.3 mM	210 ± 17 mM^−1^ min^−1^
Immobilized LAD	92.2 ± 7.8	15.5 ± 2.1 mM	240 ± 18 mM^−1^ min^−1^
Nox	340 ± 31	27.1 ± 2.3 µM	630 ± 48 µM^−1^ min^−1^
Immobilized Nox	330 ± 29	23.9 ± 1.8 µM	690 ± 56 µM^−1^ min^−1^

**Table 3:** Mixed and sequential co-immobilization of LAD and Nox on magnetic Fe_3_O_4_/GLA–APTES nanoparticles and production of l-xylulose under cofactor regeneration conditions.

Immobilization Method/System	Immobilization Yield (IY, %)		Loading (mg per g of Support)	Conversion Efficiency (%)	Relative Total Turnover Number (Folds)
	LAD	Nox			
Mixed	87.2 ± 5.3	83.8 ± 4.9	114 ± 6.8	66.5 ± 4.7	113
Sequential	91.4 ± 4.8	96.0 ± 2.6	122 ± 6.3	90.3 ± 3.2	153
Free LAD (control)	- ^a^	-	-	3.6 ± 0.3	1

^a^ Not applicable.

## Data Availability

Data are contained within the article.

## Supplementary Materials

The following supporting information can be downloaded at: https://www.mdpi.com/article/10.3390/ijms25052746/s1.
