# Supplementary material for: Sequential Co-Immobilization of Enzymes on Magnetic Nanoparticles for Efficient l-Xylulose Production
Source: Int J Mol Sci. 2024 Feb 27;25(5):2746. doi: 10.3390/ijms25052746 (PMC10932307; doi:10.3390/ijms25052746)
Supplement: Supplementary file 1 [file ijms-25-02746-s001.zip › ijms-2857857-supplementary.pdf]

Supplementary file

# Sequential Co-immobilization of Enzymes on Magnetic Nanoparticles for Efficient L-Xylulose Production

**Sanjay K. S. Patel+, Rahul K. Gupta+, Karthikeyan K. Karuppanan, In-Won Kim, and Jung-Kul Lee***

Department of Chemical Engineering, Konkuk University, Seoul-05029, Republic of Korea

+ These authors equally contributed to this work.

* Correspondence: jkrhee@konkuk.ac.kr; Tel: +82-2-450-3505; Mailing address: Department of Chemical Engineering, Konkuk University, Seoul-05029, Republic of Korea.

**Table S1**. Secondary structures analysis of free and immobilized enzymes on $Fe_3O_4$-APTES/GLA nanoparticles.

| **Enzymes** | **α-helix (%)** | **β-sheet (%)** | **β-turn (%)** | **Others (%)** |
|---|---|---|---|---|
| Free LAD | 39.1 | 31.6 | 12.1 | 17.2 |
| Immobilized LAD | 46.9 | 22.3 | 20.4 | 10.4 |
| Free Nox | 60.7 | 10.5 | 7.9 | 20.9 |
| Immobilized Nox | 71.3 | 6.2 | 10.2 | 12.3 |

**Table S2.** Influence of enzyme ratio on conversion of L-arabinitol to L-xylulose.

| Ratio (LAD:Nox) | Relative conversion (%) |
|---|---|
| 1:2 | 33.4 ± 2.7 |
| 1:3 | 35.7 ± 2.8 |
| 2:1 | 32.8 ± 3.0 |

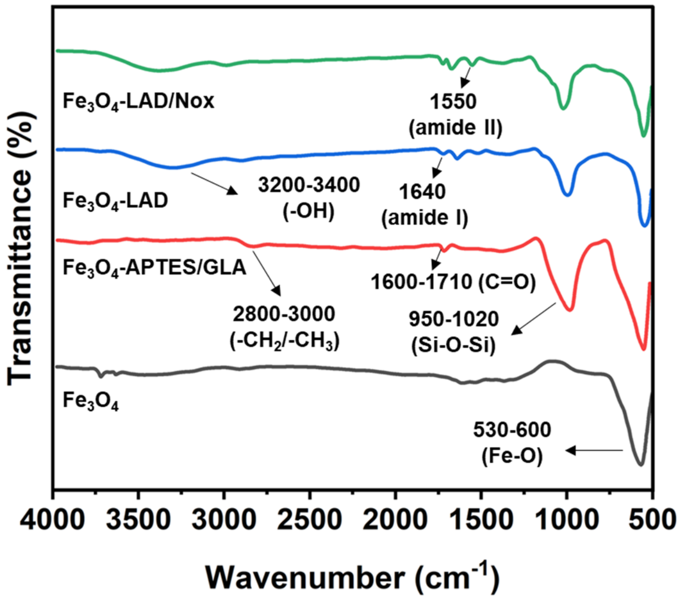


**Figure S1.** Fourier-transform infrared spectra of pure $Fe_3O_4$, functionally activated ($Fe_3O_4$-APTES/GLA), immobilized with LAD ($Fe_3O_4$-LAD), and sequentially co-immobilized LAD and Nox ($Fe_3O_4$-LAD/Nox).

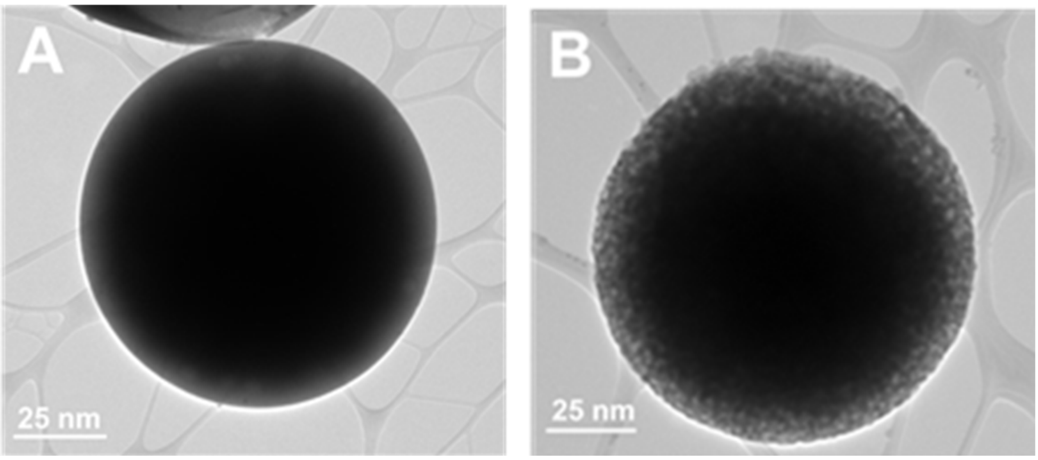


**Figure S2.** High-resolution transmission electron microscopy images of free nanoparticles (A), and with sequentially co-immobilized LAD and Nox ($Fe_3O_4$-LAD/Nox) (B).

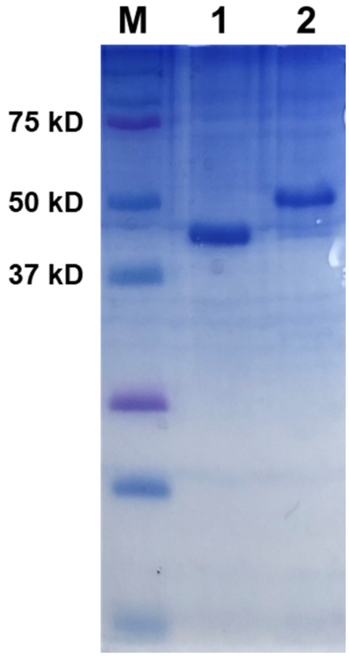


**Figure S3.** SDS-PAGE analysis. M - Protein marker, lane 1 - purified LAD, and lane 2 - purified Nox.
